# Primary Care Screening and Management of Hearing Loss in Older Adults: A Systematic Review

**DOI:** 10.7759/cureus.108230

**Published:** 2026-05-04

**Authors:** Lama B Almutairi, Ahmed Y Ayoub, Yazan Y Nahari, Hashim M Alameer, Muath H Alqesair, Abdullah S Alghamdi, Hassan A Alalawi, Rahaf K Alhajji, Refal F Alfaya, Mousa H Mawkili

**Affiliations:** 1 College of Medicine, Qassim University, Qassim, SAU; 2 College of Medicine, Jazan University, Jazan, SAU; 3 Medical School, University of Szeged Albert Szent-Györgyi Medical School, Szeged, HUN; 4 College of Medicine, Medical University of Warsaw, Warsaw, POL; 5 General Practice, Prince Saud Bin Jalawi Hospital, Al-Ahsa Health Cluster, Al-Ahsa, SAU; 6 College of Medicine, King Khalid University, Abha, SAU; 7 Family Medicine, Jazan Health Cluster, Jazan, SAU

**Keywords:** age-related hearing loss, audiology services, diagnostic accuracy, early detection, family medicine, healthcare utilisation, hearing aids, older adults, primary care, screening

## Abstract

Age-related hearing loss (presbycusis) affects a substantial proportion of older adults and is frequently encountered in family medicine and primary care settings, yet it remains significantly under-detected and under-managed despite its impact on communication, cognitive function, and quality of life. This systematic review synthesised evidence on the early detection and management of hearing loss in adults aged 55 years and older within family medicine and primary care contexts, with a focus on ear, nose, and throat (ENT)-related outcomes. A systematic search of PubMed, Google Scholar, and the Cochrane Library was conducted from 2000 to 2026 using terms related to hearing loss, presbycusis, screening, primary care, and family medicine. Eligible studies included original research, randomised controlled trials (RCTs), cohort studies, cross-sectional studies, and pragmatic trials, reporting ENT-related outcomes in primary care or community health settings. Studies were quality-appraised using design-appropriate tools: Cochrane Risk of Bias 2 (RoB 2) for RCTs, Newcastle-Ottawa Scale for cohort studies, Quality Assessment of Diagnostic Accuracy Studies 2 (QUADAS-2) for diagnostic accuracy studies, Appraisal Tool for Cross-Sectional Studies (AXIS) for cross-sectional surveys, and Risk Of Bias In Non-randomised Studies of Interventions (ROBINS-I) for non-randomised interventional studies. Of 888 records identified, 11 studies were included after deduplication and screening and were synthesised narratively, comprising a total of 25,964 participants across six countries. Screening tools, including the Hearing Handicap Inventory for the Elderly-Screening version (HHIE-S), whispered voice test, single-question screening, and smartphone-based audiometry, demonstrated moderate-to-high diagnostic accuracy when validated against pure-tone audiometry. Primary care-based electronic screening alerts increased audiology referral rates by approximately fivefold. Both conventional hearing aids and personal sound amplification devices improved self-perceived hearing function, and hearing aid use was associated with reduced emergency department visits and hospitalisations. General practitioners (GPs) demonstrated suboptimal knowledge and practices regarding age-related hearing loss screening. Overall, the evidence supports integrating structured hearing screening protocols into family medicine using validated brief tools and electronic health record alerts, while management through hearing aids and community-based amplification models is effective; however, addressing GP knowledge gaps and systemic barriers is essential to translate evidence into routine primary care practice.

## Introduction and background

Hearing loss is a common chronic sensory impairment among older adults worldwide. Approximately one-third of individuals over 65 years are affected by disabling hearing loss, and prevalence is expected to increase with population ageing [1,2]. Age-related hearing loss, known as presbycusis, is a progressive, bilateral, high-frequency sensorineural hearing loss (SNHL) caused by degeneration of cochlear hair cells and neural pathways. In addition to impairing communication, presbycusis is associated with cognitive decline, social isolation, depression, functional disability, and increased healthcare utilisation [3,4].

In older adults, the diagnosis and initial management of hearing loss often occur in primary care. General practitioners (GPs) are well-positioned to identify hearing loss due to their ongoing, patient-centred relationships and frequent clinical contact [1-5]. However, hearing loss remains substantially underdetected and under-referred in primary care, with delays between symptom onset and intervention often lasting years [3,6].

Several screening methods are available for use in primary care. These include self-report tools, bedside assessments like the whispered voice test, and technology-based approaches, such as portable audiometers and smartphone-based pure-tone audiometry [1-11]. These methods vary in accuracy, resource requirements, and feasibility within routine consultations. Despite the availability of these tools, uncertainty remains regarding the overall benefit of screening asymptomatic adults, highlighting the need for stronger evidence on screening effectiveness and outcomes [3,5,7].

Management of hearing loss in primary care extends beyond referral to audiology services. Options include conventional hearing aids, over-the-counter (OTC) devices, personal sound amplification products (PSAPs), and community-based delivery models. Evidence comparing the effectiveness of these approaches in improving quality of life, communication, and healthcare utilisation remains limited [5-11].

A further challenge is the gap in GP knowledge, attitudes, and practices related to hearing loss [2,4,7]. Many GPs report limited awareness of presbycusis, unfamiliarity with validated screening tools, and low confidence in initiating management. Addressing this gap is essential for improving early detection and care [3,5,7].

This systematic review synthesises evidence on the early detection and management of hearing loss in older adults within primary care. It focuses on ear, nose, and throat (ENT)-related outcomes, including screening accuracy, referral patterns, hearing aid uptake, functional outcomes, healthcare utilisation, and GP knowledge and practice. The aim is to inform clinical guidelines and support improvements in primary care audiology.

## Review

Methods

Search Strategy

A systematic literature search was conducted in PubMed, Google Scholar, and the Cochrane Library for studies published between January 2000 and December 2025 (Table 1). This timeframe was selected to reflect contemporary primary care practice and current hearing technologies. Search terms were grouped into three domains: hearing loss (e.g., presbycusis and SNHL), primary care (e.g., general practice and family medicine), and interventions and outcomes (e.g., screening, audiometry, referral, and hearing aids). Boolean operators were used to combine terms. No language restrictions were applied; non-English studies were assessed using available translations.

**Table 1:** Literature search strategy used in the systematic review This table summarises the databases searched, time frame, key search terms, and Boolean operators used in the systematic review. Searches were conducted in PubMed, Google Scholar, and the Cochrane Library between January 2000 and December 2025. The strategy combined terms related to hearing loss, presbycusis, primary care, family medicine, and screening using appropriate Boolean operators (AND/OR) across conceptual domains.

Database	Timeframe	Search terms (key concepts)	Boolean operators/notes
PubMed	January 2000–December 2025	Hearing loss, presbycusis, primary care, screening, and audiometry	AND/OR combinations across three domains
Google Scholar	January 2000–December 2025	Same keywords as above	Broad search for gray literature
Cochrane Library	January 2000–December 2025	Hearing loss AND screening AND primary care	Filtered for trials and reviews

Eligibility Criteria

Eligible studies included original research (randomised controlled trials (RCTs), cohort, cross-sectional, quasi-experimental, or pragmatic studies) involving participants aged 55 years or older, conducted in or applicable to primary care or community settings, and reporting at least one ENT-related outcome. Outcomes included screening accuracy, referral rates, audiological results, hearing aid use, quality of life, healthcare utilisation, or GP knowledge and practice (Table 2).

**Table 2:** Inclusion and exclusion criteria for study selection This table outlines the eligibility criteria applied during study selection for the systematic review. Inclusion criteria specified population characteristics (adults aged ≥55 years), settings (primary care or community-based), eligible study designs (randomised controlled trials, cohort studies, cross-sectional studies, quasi-experimental and pragmatic studies), and relevant ENT-related outcomes. Exclusion criteria included non-original research, non-age-related hearing conditions, paediatric populations, and studies without full-text availability.

Category	Criteria	Details
Inclusion	Population	Adults aged ≥55 years
	Setting	Primary care or community health settings
	Study design	RCTs, cohort, cross-sectional, quasi-experimental, pragmatic studies
	Outcomes	ENT-related outcomes (screening accuracy, referral, hearing aids, QoL, etc.)
	Language	No restrictions
Exclusion	Publication type	Reviews, editorials, opinion pieces, case reports, conference abstracts
	Population	Paediatric or non-age-related hearing conditions
	Data availability	Studies without original data or full text

Exclusion criteria were reviews, editorials, opinion pieces, case reports or series, studies without original data, animal studies, conference abstracts without full texts, and studies focused on paediatric or non-age-related hearing conditions.

Study Selection

Records were imported and deduplicated. Two independent reviewers screened titles and abstracts against eligibility criteria, with disagreements resolved by discussion. Full texts of potentially eligible studies were assessed in detail. The Preferred Reporting Items for Systematic Reviews and Meta-Analyses (PRISMA) 2020 flow diagram summarises the selection process [12].

Data Extraction

Data were extracted using a structured form, including study characteristics, population details, sample size, intervention or screening method, and key ENT-related outcomes. For diagnostic studies, sensitivity, specificity, predictive values, and area under the curve (AUC) were recorded. For intervention studies, effect estimates (e.g., mean differences, odds ratios, confidence intervals) were extracted. For studies on healthcare utilisation, utilisation and cost data were collected.

Quality Appraisal and Risk-of-Bias Assessment

Study quality was assessed using design-specific tools. The Cochrane Risk of Bias tool version 2 (RoB 2) was used for RCTs [13]. The Newcastle-Ottawa Scale (NOS) was applied to cohort studies [14]. Diagnostic accuracy studies were evaluated using the Quality Assessment of Diagnostic Accuracy Studies 2 (QUADAS-2) tool [15]. Cross-sectional survey studies were appraised using the Appraisal of Cross-Sectional Studies (AXIS) tool [16]. Non-randomised interventional studies were assessed using the Risk Of Bias In Non-randomised Studies - of Interventions (ROBINS-I) tool [17]. These tools evaluate domains such as selection bias, measurement validity, confounding, and reporting quality.

Results

Study Selection

**Figure 1:**
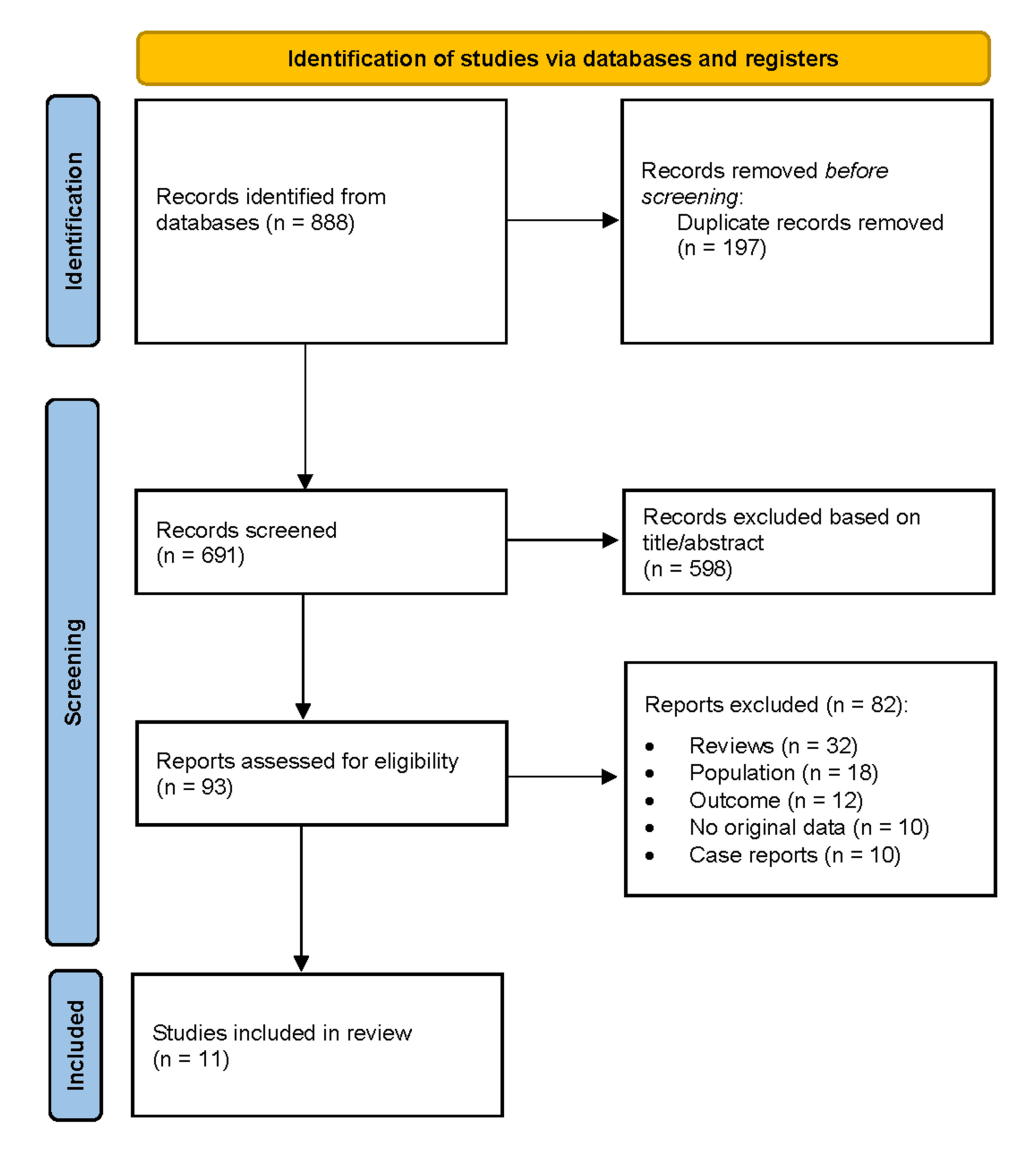
PRISMA flow diagram depicting the study selection process for the systematic review PRISMA (Preferred Reporting Items for Systematic Reviews and Meta-Analyses) flow diagram detailing the study selection process for this systematic review [12]. Following the identification of records through database searching and other sources, duplicates were removed, and the remaining records were screened. Full-text articles were assessed for eligibility, with exclusions documented along with reasons. Studies meeting the inclusion criteria were included in the final synthesis.

The search identified 888 records, with 197 duplicates removed. Of 691 records screened, 598 were excluded. Ninety-three full texts were assessed, and 82 were excluded due to ineligible design, population, or outcomes. Eleven studies met the inclusion criteria (Figure 1).

Study Characteristics

The 11 studies were conducted in six countries, most commonly the United States (n = 6) [1-6], followed by Brazil [7,8], the Netherlands [9], South Africa [10], and China [11]. Study designs included RCTs (n = 3) [1,4,5], cohort studies (n = 2) [9,11], diagnostic accuracy studies (n = 3) [7,8,10], one cross-sectional survey (n = 1) [11], and two non-randomised interventional studies [2,3]. Sample sizes ranged from 138 to 14,877 participants. All studies included adults aged ≥55 years in primary care or community settings (Table 3).

**Table 3:** Characteristics and outcomes of the included studies on the screening and management of age-related hearing loss in primary care and older adults This table summarises the key characteristics, methodologies, and ENT-related outcomes of studies included in the systematic review evaluating screening and management of age-related hearing loss in adults aged 55 years and older. Studies span randomised controlled trials, cohort studies, cross-sectional studies, diagnostic accuracy studies, pragmatic trials, and surveys conducted across multiple countries. Interventions include validated screening tools (e.g., Hearing Handicap Inventory for the Elderly-Screening version (HHIE-S), whispered voice test, single-question screening, smartphone-based audiometry), electronic health record–based referral prompts, and hearing rehabilitation strategies, such as hearing aids and personal sound amplification devices. Outcomes reported include diagnostic accuracy measures (sensitivity, specificity, area under the curve (AUC), positive predictive value (PPV), negative predictive value (NPV)), referral rates, hearing aid uptake, communication and functional outcomes, and healthcare utilisation. Abbreviations: RCT, randomised controlled trial; HHIE-S, Hearing Handicap Inventory for the Elderly-Screening version; EHR, electronic health record; BPA, Best Practice Alert; PTA, pure-tone audiometry; TBHS, telephone-based hearing screening; PSAD, personal sound amplification device; CHW, community health worker; SF-12, 12-item Short Form Health Survey; AUC, area under the curve; PPV, positive predictive value; NPV, negative predictive value; RAU, rationalized arcsine units; ED, emergency department; HA, hearing aid; OTC, over-the-counter; KAP, knowledge, attitudes, and practices; ARHL, age-related hearing loss; CI, confidence interval

Authors (Study ID)	Country	Study design	Population	Sample size	Screening/management method	Key ENT-related outcomes	Main findings
Yueh et al.[1]	USA	RCT (four-arm)	Older veterans seeking general medical care	2,305	Tone-emitting otoscope (AudioScope), HHIE-S questionnaire, combined, vs. no screening (control)	Screening positive rates: AudioScope 18.6%, HHIE-S 59.2%, combined 63.6%. Hearing aid use at one year: 6.3%, 4.1%, 7.4%, 3.3% respectively (P < 0.01 audiology evaluation rates:="">	Screening significantly increased hearing aid use. AudioScope was more efficient (lower false positive rate). Results most applicable to populations with few cost barriers.
Zazove et al. [2]	USA	Multiple-baseline design (stepped-wedge variant)	Patients aged ≥55 years at 10 family medicine clinics	14,877 eligible; 5,893 completed HHI	Electronic Best Practice Alert (BPA) in Epic EHR, prompting a single question + Hearing Handicap Inventory (HHI)	Referral rates: baseline 2.2% → 10.7% post-intervention (P < 0.001 hhi of patients). Referrals deemed appropriate. Hearing aid candidates. Mean better ear dB, worse dB. pta:="">	Electronic alert increased audiology referrals ~5-fold. Single-question screener effective in family medicine. Early-stage hearing loss detected.
Smith et al. [3]	USA	Pragmatic clinical trial (multi-site, three-arm)	Adults aged 65–75 years at six primary care clinics (family medicine and internal medicine)	660 (220 per arm)	Telephone-based hearing screening (TBHS): at-home without provider encouragement, at-home with provider encouragement, in-clinic with provider encouragement	TBHS completion: in-clinic 100%, home with encouragement 26.8%, home without 22.7%. Of 216 who failed, 48.1% completed diagnostic evaluation; 45.6% hearing aid candidates.	In-clinic screening had a fourfold higher completion rate than at-home. Provider encouragement alone not effective. In-clinic integration most effective.
Nieman et al. [4]	USA	RCT (open-label, two-group parallel)	Community-dwelling adults ≥60 years with audiometric hearing loss; low-income population	151 (78 intervention, 73 control)	Community health worker-delivered personal sound amplification device (PSAD) vs. wait-list control	HHIE-S change: intervention -13.2 vs. control +0.6 (treatment effect -12.98, 95% CI -15.51 to -10.42). SF-12 physical: +4.58 (95% CI 0.70-7.91). 90.5% used device ≥1 hour/day.	CHW-delivered PSAD improved communication function and was feasible in underserved populations.
Humes et al. [5]	USA	RCT (double-blind, placebo-controlled, three-arm)	Community-dwelling adults 55–79 years with mild-to-moderate age-related hearing loss	154	Audiology best-practice vs. OTC/consumer-decides vs. placebo hearing aids	HHIE benefit: 18.2 (AB), 12.3 (CD), 5.5 (placebo). Speech-in-noise improvement: AB 21.3, CD 26.6, P 8.7 RAU. Device use ~6-7 hours/day.	Both audiology and OTC hearing aids improved outcomes vs. placebo. OTC slightly inferior but more accessible.
Servidoni et al. [6]	Brazil	Cross-sectional	Adults >60 years (mean 71.6±8.1) at the ENT clinic	138	HHIE-S vs. pure-tone audiometry	Prevalence: HHIE-S 76.1%, PTA 79.7%. Sensitivity 89.1%, specificity 75.0%, accuracy 86.2%. PPV 93.3%, NPV 63.6%.	HHIE-S showed high sensitivity and accuracy; suitable for primary care screening.
Labanca et al. [7]	Brazil	Cross-sectional (diagnostic accuracy)	Adults 60-97 years at the geriatric centre	210 (420 ears); 42 reproducibility subset	Whispered voice test vs. pure-tone audiometry	AUC: 0.900-0.918 for key phrases. Kappa: 0.810-0.877. Hearing loss prevalence: 68.8%.	The whispered voice test is reliable, low-cost, and reproducible for primary care screening.
Oosterloo et al. [8]	Netherlands	Prospective cohort	Population-based sample (Rotterdam Study), mean age 69.6 ± 9.8	4,906	Single-question screening vs. audiometry	AUC: 0.70 (mild HL), 0.86 (moderate HL); up to 0.90 with covariates. Sensitivity: 69.9%, specificity: 69.2% (mild HL).	Single question useful for screening moderate hearing loss; limited for individual diagnosis.
Louw et al. [9]	South Africa	Cross-sectional	Adults in primary care clinics; subgroup ≥60 years	1,084 (screening); 195 (assessment)	Self-report + smartphone audiometry (hearScreen app) vs. diagnostic audiometry	Combined method: sensitivity 72.4%, specificity 100%, accuracy 81.0%. Self-report alone: sensitivity 84.3%, specificity 62.3%.	Combined self-report and smartphone audiometry highly specific; suitable for low-resource settings.
Ge et al. [10]	China	Cross-sectional survey	General practitioners in community health centres	1,022	KAP survey on ARHL screening	Knowledge: 69.90 ± 32.27, attitude: 66.09 ± 7.15, practice: 59.89 ± 21.99. Only 24.3% full knowledge.	GPs had suboptimal knowledge/practice. System-level interventions required beyond training.
Mahmoudi et al. [11]	USA	Retrospective cohort	Medicare beneficiaries ≥65 years with hearing loss	1,336 (602 HA users, 734 non-users)	Hearing aid use vs. non-use	ED visits: 2%, hospitalisations: 2%, hospital nights: 0.46. Medicare spending: $71; total spending: +$1,125.	Hearing aids reduced acute healthcare use but increased total costs due to device-related expenses.

Quality Assessment and Risk of Bias

Five studies were rated high quality [1,5,7-9], and six were of moderate quality [2-4,6,10,11]. RCTs generally showed adequate randomisation, although one open-label design introduced potential bias [4]. Cohort studies were robust overall, but one retrospective study had risks of misclassification and confounding [11]. Diagnostic studies used appropriate reference standards. The survey study reported a high response rate and a validated instrument [11]. Non-randomised studies showed a moderate risk of bias due to a lack of randomisation (Table 4).

**Table 4:** Quality appraisal and risk-of-bias assessment of studies included in the systematic review This table summarises the methodological quality and risk of bias assessment of all studies included in the systematic review of screening and management of age-related hearing loss in adults aged 55 years and older. Studies were evaluated using design-appropriate validated tools, including the Cochrane Risk of Bias tool version 2 (RoB 2) for randomised controlled trials (RCTs) [13], the Newcastle-Ottawa Scale (NOS) for cohort studies [14], the Quality Assessment of Diagnostic Accuracy Studies 2 (QUADAS-2) tool for diagnostic accuracy studies [15], the Appraisal Tool for Cross-Sectional Studies (AXIS) for survey research [16], and the Risk Of Bias In Non-randomised Studies of Interventions (ROBINS-I) tool for non-randomised interventional and pragmatic studies [17]. Risk-of-bias assessments consider key domains, including selection bias, performance bias, detection bias, attrition bias, confounding, and reporting bias, depending on study design. Overall quality ratings (high or moderate) reflect the cumulative judgement of risk of bias across domains using the respective appraisal tools. Abbreviations: RCT, randomised controlled trial; RoB 2, Cochrane Risk of Bias tool version 2; ROBINS-I, Risk Of Bias In Non-randomised Studies of Interventions; NOS, Newcastle-Ottawa Scale; QUADAS-2, Quality Assessment of Diagnostic Accuracy Studies 2; AXIS, Appraisal Tool for Cross-Sectional Studies; HHIE-S, Hearing Handicap Inventory for the Elderly–Screening version; EHR, electronic health record

Author (Study ID)	Study design	Quality appraisal tool used	Risk-of-bias summary	Overall quality
Yueh [1]	RCT	Cochrane RoB 2	Low risk of bias in randomisation and allocation concealment. Some concerns regarding blinding due to participant awareness of group assignment. Low risk of bias in outcome measurement (hearing aid use is objectively verifiable) and selective reporting. Overall, some concerns are mainly related to blinding in a pragmatic multi-arm design.	High
Zazove [2]	Non-randomised interventional (stepped-wedge design)	ROBINS-I	Some residual confounding due to potential secular time trends, although partially mitigated by the stepped-wedge design. Low selection bias, as all eligible clinic patients were included. Outcomes objectively derived from EHR data. Low reporting bias. Intervention standardised across sites.	Moderate
Smith [3]	Pragmatic clinical trial (non-randomised allocation)	ROBINS-I	Cluster-level allocation introduces confounding. Selection bias is minimised through consecutive enrolment. Objective outcome capture for screening completion. High attrition in at-home arms. Protocol prospectively registered, reducing reporting bias.	Moderate
Nieman [4]	RCT	Cochrane RoB 2	Open-label design introduces performance bias; however, the primary outcome (HHIE-S) is self-reported, reducing the impact of the lack of blinding. Adequate randomisation and allocation concealment. Low attrition and low risk of selective reporting.	Moderate
Humes [5]	RCT	Cochrane RoB 2	A double-blind placebo-controlled design minimises performance and detection bias. Strong randomisation and allocation concealment. Blinded outcome assessment. Low attrition and low risk of selective reporting.	High
Servidoni [6]	Cross-sectional (diagnostic accuracy study)	QUADAS-2	Consecutive clinic sample with some applicability concerns due to the clinical setting. Index test (HHIE-S) applied independently of the reference standard. Appropriate reference standard (pure tone audiometry). All participants received both tests.	Moderate
Labanca [7]	Cross-sectional (diagnostic accuracy study)	QUADAS-2	Low risk of bias in patient selection (consecutive sample). The index test (whispered voice test) was applied independently of the reference standard. Appropriate reference standard (pure-tone audiometry). Strong reproducibility assessment and complete flow.	High
Oosterloo [8]	Prospective cohort study	Newcastle-Ottawa Scale (NOS)	Population-based sample with low selection bias. Exposure (single-question screening) is clearly defined. Outcome measured using gold-standard audiometry. Confounders (age, sex, and education) adjusted. High methodological quality (8/9 NOS stars).	High
Louw [9]	Cross-sectional (diagnostic accuracy study)	QUADAS-2	Clinic-based sample introduces potential spectrum bias. Index tests (self-report and smartphone audiometry) were performed before the reference standard. Appropriate reference standard used. Moderate concerns regarding applicability to the older adults subgroup.	Moderate
Ge [10]	Cross-sectional survey	AXIS tool	High response rate (93.6%) with low non-response bias. Representative sampling across 51 community health centres. Validated questionnaire used. Potential social desirability bias in self-reported practice. Overall, low risk of bias.	High
Mahmoudi [11]	Retrospective cohort study	Newcastle-Ottawa Scale (NOS)	Exposure (hearing aid use) based on self-report, introducing potential misclassification. Outcomes derived from administrative claims data (objective). Residual confounding possible due to unmeasured socioeconomic and behavioural factors. Moderate methodological quality (6/9 NOS stars).	Moderate

Screening Tools and Diagnostic Accuracy

The HHIE-S demonstrated high sensitivity but lower specificity [1,7]. The whispered voice test showed strong diagnostic accuracy and reproducibility, with high AUC values and no resource requirements [8]. A single self-report question had moderate accuracy, with higher specificity for severe hearing loss [9,10]. Smartphone-based pure tone audiometry, combined with self-report, improved accuracy and specificity in primary care [10].

Primary Care Screening Interventions and Referral Outcomes

Two studies evaluated structured interventions. Integration of electronic health record (EHR) prompts increased audiology referral rates, with many patients identified as candidates for hearing aids [2]. A trial of telephone-based hearing screening (TBHS) found higher completion and follow-up rates with in-clinic delivery compared to home-based approaches [3].

Management Outcomes

Four studies examined management. Screening increased hearing aid uptake and improved communication outcomes [1]. Both audiology-based and OTC hearing aid models improved hearing-related function and speech outcomes [5]. Community health worker-led provision of personal sound amplification devices improved communication and showed high adherence in underserved populations [4].

Healthcare Utilisation Outcomes

Hearing aid use was associated with reduced emergency department visits, hospitalisations, and hospital nights [6]. Although overall healthcare costs increased due to device-related expenses, reduced acute care use suggests potential long-term economic benefit.

General Practitioner Knowledge, Attitudes, and Practice

A cross-sectional survey of GPs found moderate knowledge and practice scores, with gaps in awareness and use of screening tools [11]. Some GPs did not recognise age-related hearing loss as a medical condition. Higher knowledge was associated with better practice, but system-level changes were needed to improve care delivery.

Limitations

This review has several limitations. Most included studies were conducted in high-income countries, particularly the United States, which may limit generalisability to other settings. Considerable heterogeneity in study design, populations, screening methods, and outcomes prevented meta-analysis, requiring a narrative synthesis. Some studies used clinic-based samples, introducing potential selection and spectrum bias. The retrospective design of Mahmoudi et al. [11] limits causal inference due to possible residual confounding. Follow-up periods in intervention studies were generally short, making long-term outcomes uncertain. In addition, evidence on GP knowledge and practice was limited to a single study, reducing broader applicability.

## Conclusions

Hearing loss in older adults is common, frequently underdiagnosed, and manageable within family medicine. Brief screening tools can be incorporated into routine primary care, particularly when supported by structured workflows and digital systems. Multiple models of hearing aid provision, including clinic-based and lower-cost approaches, improve hearing-related quality of life and communication. Gaps in GP knowledge and practice persist, and system-level strategies appear more effective than education alone in improving screening uptake. Family medicine has an important and underused role in addressing untreated hearing loss, and strengthening primary care-based audiology services is clinically warranted.
